# Multiple signaling pathways in Sertoli cells: recent findings in spermatogenesis

**DOI:** 10.1038/s41419-019-1782-z

**Published:** 2019-07-17

**Authors:** Fei-Da Ni, Shuang-Li Hao, Wan-Xi Yang

**Affiliations:** The Sperm Laboratory, College of Life Sciences, Zhejiang University, 310058 Hangzhou, Zhejiang China

**Keywords:** Cell signalling, Spermatogenesis

## Abstract

The functions of Sertoli cells in spermatogenesis have attracted much more attention recently. Normal spermatogenesis depends on Sertoli cells, mainly due to their influence on nutrient supply, maintenance of cell junctions, and support for germ cells’ mitosis and meiosis. Accumulating evidence in the past decade has highlighted the dominant functions of the MAPK, AMPK, and TGF-β/Smad signaling pathways during spermatogenesis. Among these pathways, the MAPK signaling pathway regulates dynamics of tight junctions and adherens junctions, proliferation and meiosis of germ cells, proliferation and lactate production of Sertoli cells; the AMPK and the TGF-β/Smad signaling pathways both affect dynamics of tight junctions and adherens junctions, as well as the proliferation of Sertoli cells. The AMPK signaling pathway also regulates lactate supply. These signaling pathways combine to form a complex regulatory network for spermatogenesis. In testicular tumors or infertile patients, the activities of these signaling pathways in Sertoli cells are abnormal. Clarifying the mechanisms of signaling pathways in Sertoli cells on spermatogenesis provides new insights into the physiological functions of Sertoli cells in male reproduction, and also serves as a pre-requisite to identify potential therapeutic targets in abnormal spermatogenesis including testicular tumor and male infertility.

## Facts

- Sertoli cells support, nourish, and protect spermatogenic cells via various signal pathways.
- The TGF-β/Smad, AMPK, and MAPK signaling pathways in Sertoli cells support spermatogenesis via regulating cell junction dynamics, proliferation of Sertoli cells and germ cells, and lactate supply for spermatids.
- Activity of the TGF-β/Smad, AMPK, and MAPK signaling pathways in Sertoli cells turns abnormal in non-obstructive azoospermia and patients with testicular cancer.

## Open questions

- Which pathway plays a decisive role when the TGF-β/Smad, AMPK, and MAPK signaling pathways in Sertoli cells regulate the same process?
- What are the detailed molecular downstream mechanisms and interactions of proteins involved in the pathways mediating physiological functions of Sertoli cells?
- What is the key role of the TGF-β/Smad, AMPK, and MAPK signaling pathways in tumorigenesis and infertility?
- Is it possible to identify specific pathways and related proteins as diagnostic and therapeutic targets for testicular cancer and male infertility?

## Introduction

Spermatogenesis is a significant physiological process of sperm production in the epithelium of the seminiferous tubules^1^. In this process, spermatogonial stem cells (SSCs) are triggered to produce spermatogonia, which will transform to spermatocytes, spermatids, and finally mature spermatozoa^2,3^. The migration of germ cells (GCs) and the release of spermatozoa require timely cell junctions disassemble and reassemble between Sertoli cells-Sertoli cells (SCs-SCs) and SCs-GCs^4,5^. Such adherens junctions (AJs) are named as ectoplasmic specializations (ES) in the testis, and are divided into the basal ES at the SCs-SCs interface and the apical ES at the SCs-spermatids interface^6^. In the mammalian testis, the basal ES, desmosomes, gap junctions, and tight junctions (TJs) between SCs form the blood–testis barrier (BTB)^7^. The TJs at the BTB are constituted of various tight junctional proteins, including the claudin (CLDN) family, junctional adhesion molecule (JAM) family, etc. (for reviews, see ref. ^8^), which will bind to actin via the zonula occludens-1, -2 and -3 (ZO-1, ZO-2 and ZO-3) in SCs^9^. At stage VII-VIII of the epithelial cycle, the preleptotene and leptotene spermatocytes must move through the BTB and enter the adluminal compartment^10^. Most researchers focused on the synchronization of spermatogenesis currently, but few of them have addressed the issue from the perspective of Sertoli cells^11–17^.

Sertoli cells are the only somatic cells in the seminiferous epithelium^18^. Throughout mammalian spermatogenesis, SCs provide morphogenetic support via cell–cell interactions and also biochemical components via secreting lactate, cytokines, and hormones^19,20^. Apart from the mechanical and nutritional support, SCs also form an immune-protective environment to protect germ cells via the BTB^21–24^. At the end of spermatogenesis, AJs between GCs and SCs allow SCs endocytosis of the elongated spermatids’ cytoplasm, and finally morphologically shape the spermatids^25^. Therefore, SCs are considered as nurse like cells to support spermatogenesis^26^.

**Fig. 1:**
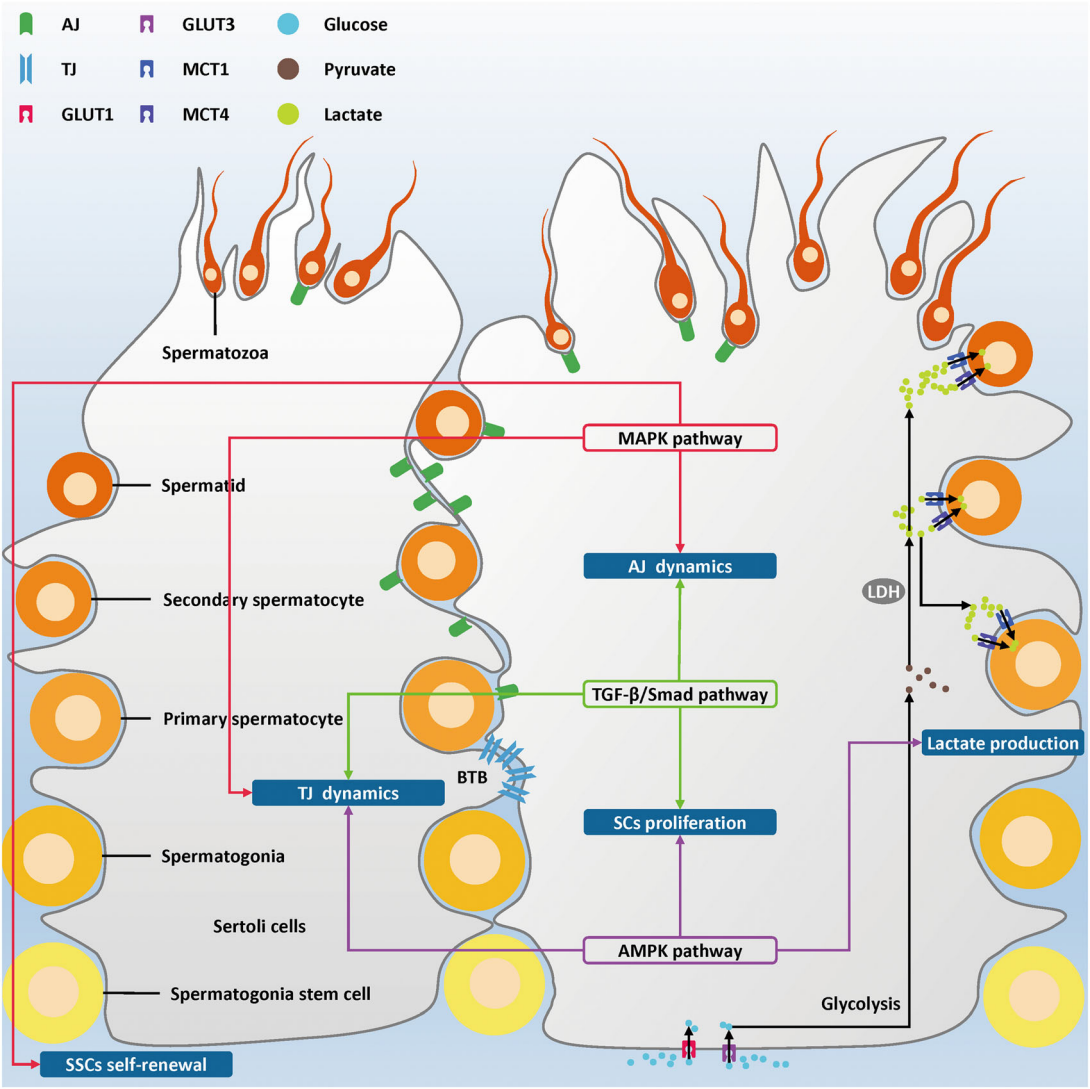
Schematic diagram illustrating the integrated influence of the TGF-β/Smad, AMPK, and MAPK signaling pathways in SCs on spermatogenesis. The TGF-β/Smad signaling pathway regulates spermatogenesis via controlling AJ, TJ dynamics, and Sertoli cell proliferation (green). The AMPK signaling pathway regulates TJ dynamics, lactate production, and Sertoli cell proliferation (purple). The MAPK signaling pathway influences AJ, TJ dynamics, and spermatogonia stem cell self-renewal, and ultimately supports spermatogenesis (red)

Accumulating studies have indicated that various signaling pathways in SCs are implicated with spermatogenesis^27,28^. Until now, numerous signaling pathways have been found in Sertoli cells, including the androgen-signaling pathway^29,30^, the AMP-activated protein kinase (AMPK) signaling pathway^31^, the follicle stimulating hormone (FSH)/adenylate cyclase/cyclic adenosine monophosphate (cAMP)/protein kinase A (PKA) signaling pathway^32^, the Hippo signaling pathway^33,34^, the intergrin mediated signaling pathway^4,35^, the Janus kinase/signal transducer and activator of transcription signaling pathway^36,37^, the mitogen-activated protein kinases (MAPK) signaling pathway^38–44^, the nuclear factor kappa B signaling pathway^45,46^, the nitric oxide/soluble guanylyl cyclase/cyclic guanosine monophosphate/protein kinase G signaling pathway^47,48^, the Notch signaling pathway^49^, the phosphatidylinositol-4,5-bisphosphate 3-kinase (PI3k)/AKT serine/threonine kinase (Akt) signaling pathway^50,51^, the Sonic Hedgehog signaling pathway^52,53^, the transforming growth factor-β (TGF-β)/Smad signaling pathway^54^, and the Wnt signaling pathway^55,56^ (Table 1). Among all these signaling pathways, the TGF-β/Smad, AMPK, and MAPK signaling pathways have attracted much more attentions in the past decade. In this review, we aim to summarize the impact mechanisms of these three pathways in SCs on spermatogenesis (Fig. 1). These three signaling pathways play dominant functions in SCs, which support the spermatogenesis via jointly affecting SCs proliferation, AJ and TJ dynamics. Moreover, the MAPK and AMPK signaling pathway affect lactate supply in SCs, while the MAPK signaling pathway also occupies a dominant position in regulating SSCs self-renewal.

**Table 1:** Roles of Sertoli cells signaling pathways in spermatogenesis

Signaling pathways	Signal molecules or environmental conditions	Species	Function	Targets	References
The AMPK pathway	17β-estradiol	Boar	Inhibiting SCs proliferation	mTORC1, p27, p53, Skp2	^100^
	Adenosine	Rat	Promoting lactate secretion in SCs	GLUT1, LDH, MCT4	^91^
	Adenosine	Rat	Regulating tight junction	ZO-1	^91^
	AIACR	Rat	Promoting lactate secretion in SCs	GLUT1, GLUT3, MCT1, MCT4	^83^
	A76	Rat	Inhibiting SCs proliferation	Raptor, p70S6K, CDKI	^99^
	Glucose deprivation	Rat	Maintaining lactate secretion in SCs	GLUT1	^92^
	Hyperthermia	Pig	Regulating tight junction	Claudin 11, JAMA, occludin, ZO-1	^96^
The classical testosterone pathway	Testosterone	Rat	Presumably supporting endocytosis of spermatid cytoplasm	Picalm, Eea1, Stx5a	^161^
The ERK pathway	FGF-2	Rat	Promoting lactate secretion in SCs	LDH	^127, 128^
	FGF-2	Rat	Presumably promoting iron supply	Transferrin	^127, 128^
	FSH	Rat	Promoting proliferation of SCs 5 days after birth	Cyclin D1	^124^
	IL-6	Rat	Disrupting BTB integrity	β-catenin	^162^
	Ouabain	Rat	Stimulating proliferation of SCs	Cyclin D1	^126^
	TGF-β3	Mouse	Presumably regulating the apical ES and BTB dynamics	JAM-B	^75^
	bFGF	Mouse	Promoting self-renewal of spermatogonia stem cells	GDNF	^123^
The FSH/AC/cAMP/PKA pathway	FSH	Mouse	Inhibiting apoptosis of SCs	Fatty acid amide hydrolase(FAAH)	^163^
	FSH	Mouse	Promoting meiosis of spermatocytes	Nociceptin	^164, 165^
The intergrin mediated pathway	Endogenous testosterone	Rat	Disrupting the apical ES	ERK	^4^
	AF-2364	Rat	Disrupting SCs-GCs anchoring junction	ERK	^35^
The JAK/STAT pathway	IL-6(interleukin-6)	Rat	Presumably proliferation of SCs	c-fos, junB, c-myc	^37^
	IFN-γ(interferon-γ)	Rat	Presumably proliferation of SCs	c-fos	^36, 37^
	Leukemia inhibitory factor (LIF)	Rat	Presumably proliferation of SCs	c-fos, AP-1	^37^
The JNK pathway	TGF-β3	Mouse	Presumably regulating the apical ES and BTB dynamics	JAM-B	^75^
	TNF-α	Mouse	Presumably regulating cell adhesion	ICAM-1	^108^
	CdCl_2_	Rat	Inhibiting CdCl_2_ induced BTB damage	α_2_-MG	^111^
The NF-κB pathway	17β-estradiol	Rat	Improving proliferation of SCs	CCND1	^166^
	TNF-α	Mouse, rat	Inducing apoptosis of GCs	FasL	^167, 168^
	TNF-α	Rat	Presumably increasing Testosterone response	Androgen receptors (AR)	^169, 170^
The NO/sGC/cGMP/PKG pathway	NO	Rat	Disturbing tight junction assembly	Occludin	^48^
	NO	Rat	Perturbing adherens junction dynamics	CDH/CATNB	^171^
The non-classical testosterone pathway	Testosterone	Hamster	Promoting glucose uptake	COX2	^172^
	Ouabain	Rat	Influencing tight junction stabilization in a dose-dependent manner	Claudin 11, connexin 43	^173^
The Notch pathway	JAG/DELTA	Mouse	Disturbing self-renewal of spermatogonia stem cells	GDNF	^119– 122^
	JAG/DELTA	Mouse	Disturbing self-renewal of spermatogonia stem cells	CYP26B1	^119, 120^
The p38 MAPK pathway	IL-1α	Mouse	Presumably regulating tight junction and adherens junction dynamics	JAM-B	^74^
	Glucose deprivation	Rat	Maintaining lactate secretion in SCs	GLUT1	^92^
	TGF-β3	Rat	Disrupting tight junction and BTB stabilization	Occludin, ZO-1, N-cadherin, claudin-11	^102, 104, 105^
	TNF-α	Rat	Disrupting adherens junction and BTB dynamics	Occludin, ZO-1, and N-cadherin	^106^
The PI3k/Akt pathway	FSH	Rat	Promoting SCs proliferation	mTOR, p70S6K, PRAS40	^99^
	3, 3′, 5-triiodothyronine (T3)	Rat	Inhibiting SCs proliferation	CyclinA2, cyclinD1, cyclinE1, PCNA, Skp2, p27	^174^
	rpS6	Rat	Perturbing tight junction	claudin-11	^7^
The Sonic Hedgehog pathway	Hedgehog	Mouse	Presumably regulating SCs-GCs interaction	WD (tryptophan-aspartate) repeat and SOCS box-containing 2 (Wsb2)	^53^
The TGF-β/Smad pathway	Activin	Mouse	Supporting SCs maturation	Gja1, Serpina5	^54^
	TGF-β2	Mouse	Presumably regulating tight junction and adherens junction dynamics	JAM-B	^74^
	TGF-β3	Mouse	Presumably regulating the apical ES and BTB dynamics	JAM-B	^75^
The Wnt noncanonical cell polarity pathway	Wt1 (Wnt4 mediated)	Mouse	Maintaining polarity of SCs	Par6b and E-cadherin	^56^
The Wnt/β-catenin pathway	Wnt	Mouse	Presumably disturbing self-renewal of spermatogonia stem cells	GDNF	^117^
	Wnt3	Mouse	Supporting establishment of gap junction between SCs and GCs	Connexin43	^175, 176^

## The TGF-β/Smad signaling pathway

**Fig. 2:**
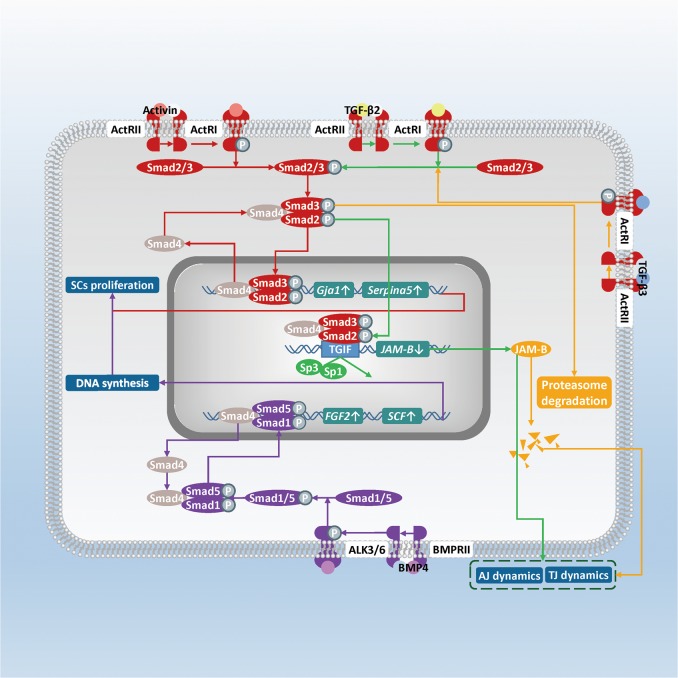
Schematic diagram illustrating the influence of the TGF-β/Smad signaling pathway in SCs on spermatogenesis. Members of TGF-β family activate the pathway via binding to TGFβRII and recruiting TGFβRI. Different TGF-βs bind to their corresponding receptor complexes, but all activated TGFβRI can phosphorylate members of Smad family, which are divided into 3 subfamilies including the receptor-regulated Smads (R-Smads, Smad1, 2, 3, 5, 8), the common Smad (Co-Smad, Smad4), and the inhibitory Smads (I-Smads, Smad6, 7). After TGFβRI phosphorylates R-Smad, R-Smad will detach from TGFβRI and bind to Smad4. Then the heteromeric complex translocates into the nucleus, binds to the promoter region and alters transcription of target gene with co-factors. (Red) Activin binds to type IIA activin receptor to activate the activin/Smad2/Smad3 pathway, then expression levels of *Gja1* and *Serpina5* rise to affect SCs maturation. BMP4 activates the BMP4/Smad1/Smad5 pathway via binding with BMPRII, and then promotes DNA synthesis and SCs proliferation (purple). Both TGF-β2 and TGF-β3 will inhibit the *JAM-B* expression level. TGF-β3 lowers the JAM-B protein level via activating TGF-β3/Smad2/Smad3 pathways to induce the ubiquitin–proteasome degradation (orange). TGF-β2 activates Smad3 (green). Then Smad3 can compete with Sp1 and Sp3 and inhibit the *JAM-B* transcription

The transforming growth factor-β (TGF-β) superfamily contains activin, inhibin, bone morphogenetic proteins (BMPs), growth and differentiation factors (GDFs), and TGF-β homodimeric proteins^57–59^. Smad4 serves as a crucial mediator of upstream signals in the TGF-β/Smad signaling pathway upon TGF-βs stimulation. Although Smad1, 3, 5, 6, 7, 8 express discriminately at birth, stages V, VII, VIII, XV, and adult based on immunohistochemical detection and RT-PCR results, Smad4 are distributed within SCs at all ages in mice and domestic fowl^60–63^. When *Smad4* was conditionally deleted in mouse Sertoli cells, the fertility of mutant mouse was impaired with smaller testis size and decreased sperm production at adult^64^. The various trends of *Smad* expression and deleted phenotype demonstrate that the TGF-β/Smad signaling pathway occupies a continuous crucial position for SCs function during spermatogenesis, while different members of the TGF-β superfamily may perform their functions at different stages (Fig. 2).

### SCs proliferation

Precisely regulated Smad2/3 signaling is required for SCs to differentiate from the proliferating state to a differentiated state. Type IIA activin receptor exhibits high-expression transiently in rat SCs at stage VII-IX^65,66^. Consistent with this, Itman et al. found that activin-induced nuclear accumulation of Smad2 and Smad3 in post mitotic mouse SCs, and also screened out the activin target genes *Gja1* and *Serpina5* via microarray analysis. These two genes encode connexin 43 and serine protease inhibitor, respectively, which are required for SCs maturation^54^. In Smad3^−/−^ mouse, delayed SCs differentiation and decreased testis size were accompanied by an inhibition of androgen receptor and Smad2 expression^67^. Uncovering the link between Smad2/3 and their downstream network may make us benefit in studying the effects of SCs proliferation on sperm output.

BMP4 and BMP6 promote proliferation and DNA synthesis of human SCs via an autocrine pathway^68,69^. BMP4 was observed to increase Smad1/5 phosphorylation and to enhance proliferation of human SCs, but administration of noggin, the BMP4 antagonist, showed conversely inhibitory effects. When Hai et al. knocked down BMP4 in human SCs, fibroblast growth factor-2 (FGF-2) and SCF production was also suppressed^70^. However, the contribution of Smad1/5 pathway against the ID2/3 pathway in BMP4-induced SCs proliferation enhancement was not evaluated in their research. This problem also exists in the study of BMP6, particularly, whether the Smad2/3 signaling pathway directly mediates the BMP6-induced proliferation, and increased levels of SCF and Glial cell-derived neurotrophic factor (GDNF) in human SCs^71^.

### TJs and AJs dynamics

TGF-βs and GDF9 participate in the regulation of TJs and AJs dynamics. Given that the transition and relocation of spermatocytes through the BTB require coordinated disassembly and reassembly of cell junctions, timely regulation of JAM-B expression is crucial for migration of GCs^72,73^. Both TGF-β2 and TGF-β3 downregulate the expression of JAM-B via the Smad3 signaling pathway. Wang and Lui discovered that in mouse SCs, TGF-β2 served as an anti-expression factor of JAM-B at the pre-transcriptional level. TGF-β2 increases phosphorylation level of Smad3, which would compete with the transcription factors Sp1 and Sp3 for the TG interacting factor (TGIF) motif, and ultimately repress the JAM-B transcription^74^. However, TGF-β3 treatment can decrease the JAM-B protein level at a post-translational way in mouse SCs. The degradation of JAM-B can be relieved upon administration of proteasome inhibitors, including MG-132 and lactacystin. This process requires Smad3/4 activation. If Smad3 and Smad4 are knocked down, TGF-β3-induced JAM-B degradation will be inhibited in turn^75^. Consequently, TGF-β2 and TGF-β3 may establish a precise-regulating network for disassembly and assembly of the BTB via the Smad signaling pathway.

Apart from JAM-B, Smad3 also supports preleptotene spermatocytes translocation by decreasing *CLDN11* expression in mouse TM4 cell lines^76^. As a component of TJ, CLDN11 is crucial for spermatocyte migration through the BTB into the adluminal compartment^77–79^. When the Smad signaling pathway is stimulated in TM4 cells, Smad3/4 binds to the GATA/NF-Y motif in *CLDN11* promoter. Quantity of the complex formed in this way will be decreased upon anti-Smad3 antibody treatment in the electrophoretic mobility shift assay. Ulteriorly, the binding complex can recruit histone deacetylase 1 and co-repressor mSin3A. Thus, transactivation of GATA and CREB, as well as the activity of the promoter in *CLDN11* gene were inhibited^76^.

Few researches have addressed the issue on the GDF9/Smad signaling pathway, but Nicholls et al. did detect disruption of the inter-Sertoli TJ permeability barrier after adding recombinant GDF9 in mouse SCs cultures^80^. GDF9 receptor ALK5 and Smad2/3 were highly detected in adult alpaca and cat SCs^81,82^. Here, we suggest that further experimental investigations should focus on whether GDF9 regulates TJs via the GDF9/Smad2/3 signaling pathway.

### The AMPK signaling pathway

**Fig. 3:**
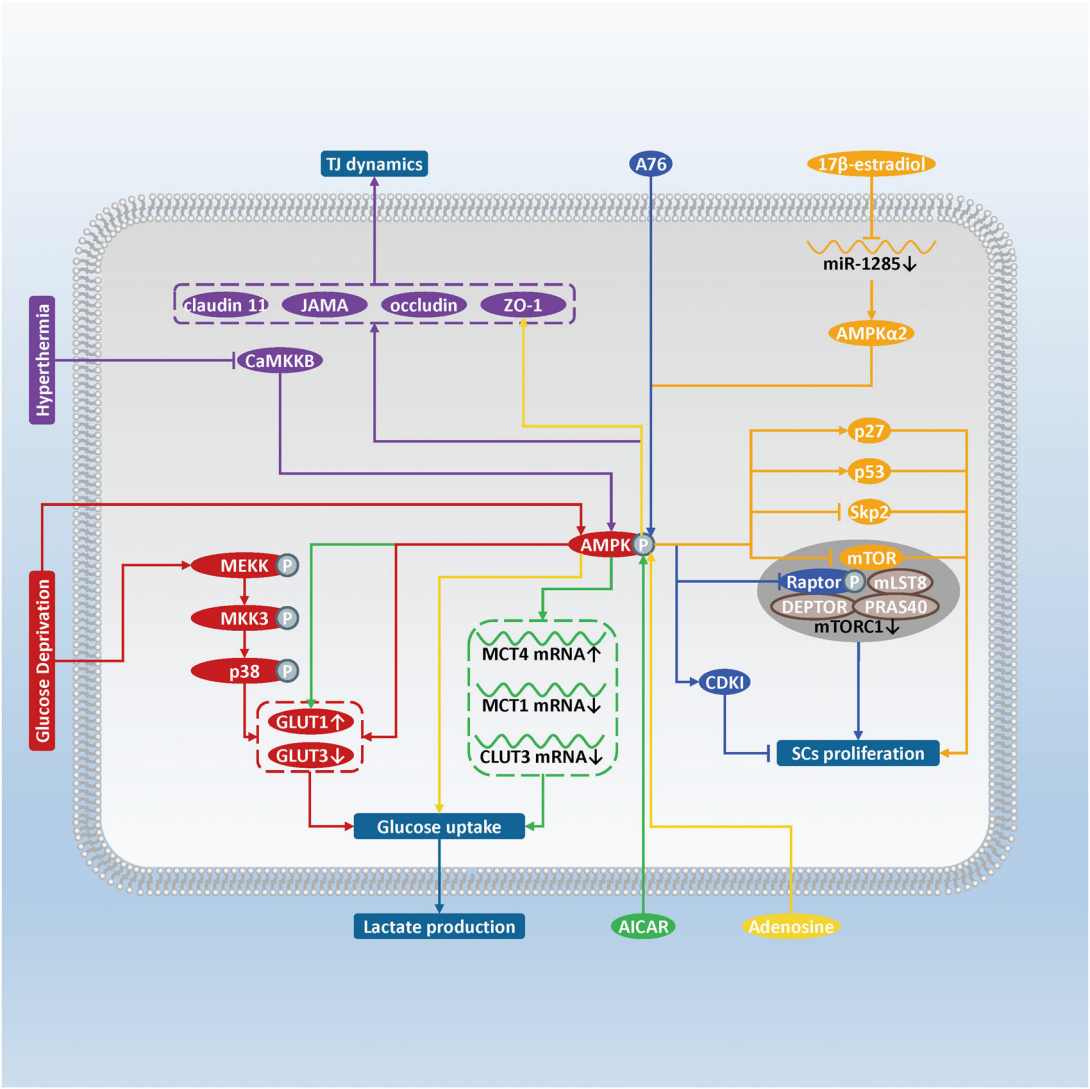
Schematic diagram illustrating the influence of the AMPK signaling pathway in SCs on spermatogenesis. AMPK is sensitively stimulated by the rise of intracellular AMP: ATP ratio, while phosphorylation induced by upstream AMPK kinases (i.e., liver kinase B1 [LKB1] and CaMKKB) and AMP binding will also stimulate AMPK. Glucose deprivation activates the AMPK and p38 MAPK pathways to upregulate GLUT1 but downregulate GLUT3 (red). Activation of the AMPK signaling pathway induced by adenosine analog AICAR increases *GLUT1* expression and MCT4 mRNA, but decreases MCT1 and GLUT3 mRNA, thus promoting glucose uptake and lactate secretion (green). Adenosine also promotes lactate secretion and stabilizes ZO-1 on SCs membrane via activating the AMPK pathway (yellow). Hyperthermia inhibits CaMKKB to block the AMPK pathway, and, therefore, affects TJ protein expression (purple). AMPK activates CDKI but phosphorylates Raptor to inhibit mTORC1, thus blocking the SCs proliferation (blue). 17β-estradiol inhibits *miR-1285* expression to maintain α2AMPK level (orange). This way, activation of AMPK is retained, which upregulates *p53* and *p27* expression but downregulates *mTOR* and *Skp2* expression, resulting finally in the reduction of the SCs number

The AMPK is a kind of heterotrimeric Ser/Thr kinase, which serves as the sensitive energy sensor and cellular energy metabolism regulator in Sertoli cells^19,83^. The AMPK signaling pathway in SCs has been found to regulate energy metabolism, junctional complex stability, and proliferation^84^. Once the balance is disrupted, the microenvironment of testis and the quality of sperm will be affected. For example, in α1AMPK globally knocked out mouse, spermatozoa showed abnormal head, curved sheaths, and impaired mobility^85^. When α1AMPK is conditionally knocked out in mouse SCs, the mutant mice still showed an abnormal phenotype, including thin head spermatozoa, reduced expression of junctional proteins (β-catenin, vimentin, occludin and ZO-1), and deregulation of energy homeostasis^86^. These findings support the contribution of the AMPK signaling pathway in SCs during spermatogenesis (Fig. 3).

### Lactate production

Lactate is a preferring energy source of spermatocytes and spermatids, the majority of which is provided by Sertoli cells^87,88^. SCs will actively convert glucose mainly into lactate. In this process, glucose transporters (GLUTs) regulate glucose metabolism via limiting substrate transmembrane transport, while monocarboxylate transporters (MCTs) control lactate transport and supply to GCs, both of which contribute to adjust lactate production in SCs^83,89,90^.

In response to various signaling factors and environmental conditions, the AMPK signaling pathway in SCs serves as a key regulator in providing lactate for energy metabolism of GCs and maintaining spermatogenesis^91^. Glucose deprivation in rat SCs will induce activation of the AMPK and p38 MAPK signaling pathway, increase the mRNA level of GLUT1 and maintain the uptake of glucose. Such adaptation ensures or rescues lactate production even in the absence of glucose^92–94^. Adenosine and its analog AICAR were also proven to promote lactate secretion from rat SCs via AMPK activation, while mechanism of AIACR regulation is illustrated more integrally^83,91^. AICAR can increase lactate production via the AMPK-induced glucose intake in rat SCs, at least through increase in GLUT1 protein level and MCT4 mRNA level, and decrease in MCT1 and GLUT3 mRNA levels^83,95^. Overall, adaptation to the environment and response to those signal molecules via the AMPK signaling pathway in SCs will thus stabilize an appropriate lactate supply for GCs energy demand.

### TJs and AJs dynamics

The AMPK signaling pathway maintains junctional complex stabilization in testis. It has been shown that activation of AMPK by adenosine stabilizes ZO-1 on rat SCs membranes, and the AMPK inhibitor compound C can decline adenosine affected ZO-1^91^. Also, heat stress can cause dysfunction of TJs in porcine testis reversibly via Ca^2+^/calmodulin-dependent protein kinase kinase B (CaMKKB) induced inhibition of the AMPK signaling pathway. Yang et al. treated SCs from 3-week-old piglets at 43 °C for 0.5 h, and such hyperthermia inhibited the AMPK signaling pathway to inhibit expression of CLDN11, JAMA, occludin, especially ZO-1 in porcine SCs^96^.

As for AJs, the relationship has been clarified between the 26S proteasome inhibitor bortezomib, the AMPK signaling pathway and AJs among SCs and GCs in mouse. Bortezomib can induce AMPK activation and then antagonize Akt and extracellular signal-regulated kinase (ERK) signaling pathway in mouse SCs. As a consequence, AJs impairment, immature GCs desquamation and sperm quantity reduction are followed^97^. Based on this phenomenon observed in bortezomib exposure, we suggest that the detailed mechanisms of the normal situation are also worth studying.

### SCs proliferation

Apart from regulating AJs integrity, SCs proliferation inhibition is also mediated by the AMPK signaling pathway^98^. AMPK activation potentially leads to detention of rat SCs proliferation at least partially by inhibition of mTORC1 and stimulation of cyclin-dependent kinase inhibitors expression. Moreover, lower activity of mTORC1 was due to accumulation of phosphorylated Raptor^99^. Consequently, SCs mitotic activity, which is stimulated by FSH and mediated by the PI3K/Akt signaling pathway, is counteracted by the AMPK signaling pathway. Similarly, the activated AMPK signaling pathway also mediated 17β-estradiol inhibition on boar SCs proliferation, which would be abolished by compound C treatment. Zhang et al. administrated 10 μM of 17β-estradiol on boar SCs and observed inhibition of miR-1285 expression^59^. Recently, they clarified that miR-1285 can downregulate α2AMPK mRNA and protein level. 17β-estradiol treatment retains AMPK activity by maintaining α2AMPK^31^. As for the downstream effect, the phosphorylated AMPK increases the expression of the cyclin-dependent kinase inhibitor p27 (p27) and tumor suppressor p53 (p53), but inhibits the protein level of phosphorylated mTOR and S-phase kinase-associated protein 2 (Skp2)^100^. This regulatory network ultimately leads to reduction of SCs number and sperm production in boars.

## The MAPK signaling pathway

**Fig. 4:**
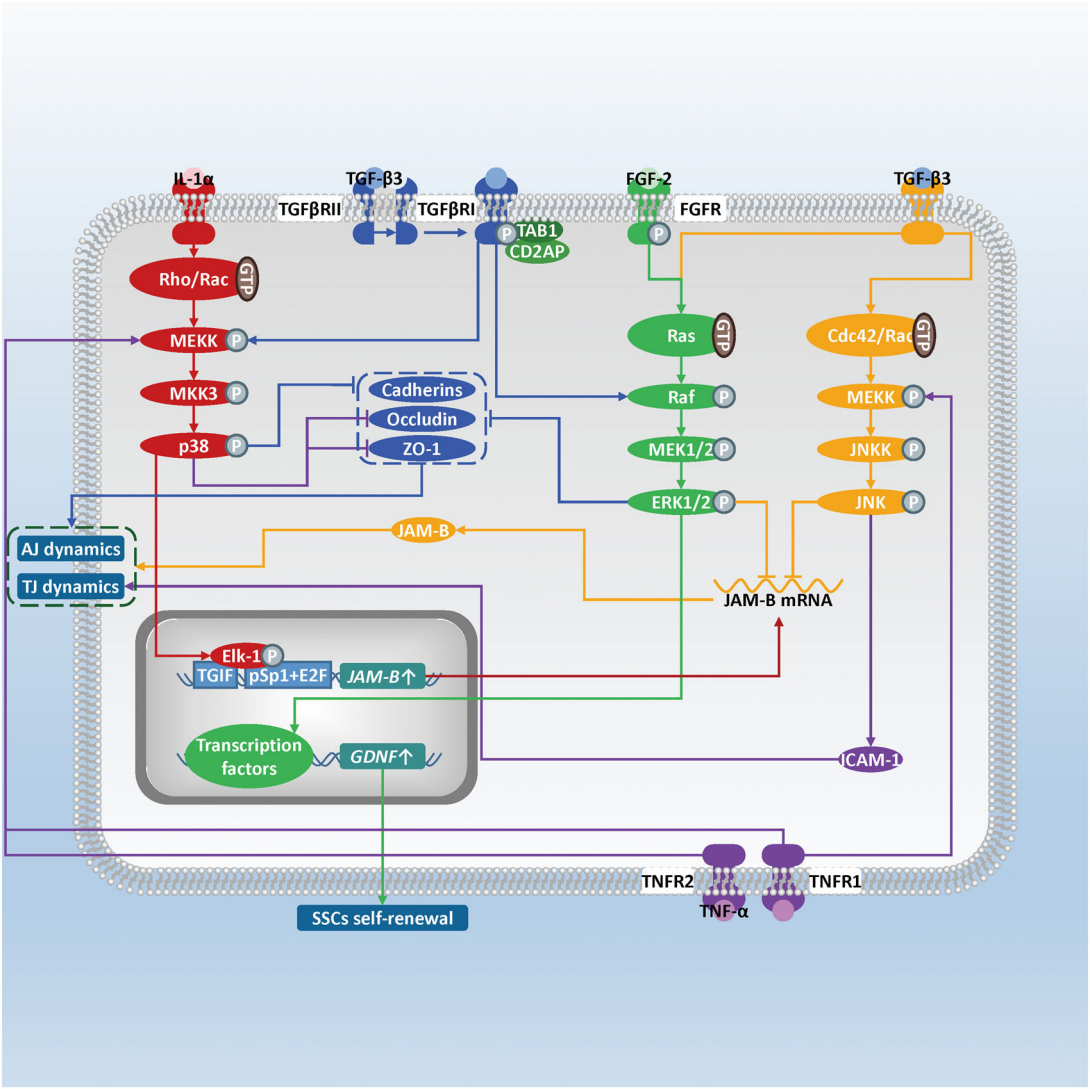
Schematic diagram illustrating the influence of the MAPK signaling pathway in SCs on spermatogenesis. MAPKs consist of JNKs, ERKs, and p38 MAPKs. After MAPKKK is activated by the signal, MAPKK and then MAPK are activated via phosphorylation. The activated MAPK will then phosphorylate its substrates. IL-1α activates via the p38 MAPK pathway, then the phosphorylated p38 MAPK phosphorylates Elk-1 and allows Elk-1 to bind onto TGIF and pSp1 + E2F motifs, which thus stimulates *JAM-B* transcription (red). Activation of the ERK and JNK pathways induced by TGF-β3 will promote JAM-B mRNA destabilization (orange). When TAB1 and CD2AP both interact with TGF-β3-TGFβRI, the activated p38 MAPK and ERK pathways will downregulate expression of occludin, ZO-1 and cadherin, and disturb SCs-GCs AJs and BTB (blue) . TNF-α administration will decrease occludin and ZO-1 via the p38 MAPK pathway but also increase ICAM-1 via the JNK pathway, thus regulating the AJ and TJ dynamics (purple). FGF-2 activates the ERK pathway to stimulate GDNF expression, thus enhancing SSCs self-renewal (green)

MAPKs belong to the Ser/Thr kinase family. There are three major subfamilies of MAPKs, i.e., c-Jun N-terminal kinase (JNK), ERK, and p38 MAPK (MAPK14). The isoforms and distribution of JNKs, ERKs and p38 MAPKs present in mammalian SCs have been summarized. In rat testis, (phosphorylated) ERK1/22, (phosphorylated) JNK1/2, (phosphorylated) p38 MAPK are located in SCs, while ERK7, JNK3 are investigated in testis (for reviews, see ref. ^1^). According to the microarray data, a majority of MAPK pathway-related genes exist in immature rat SCs, which shows the existence of the MAPK signaling cascades in SCs^101^ (Fig. 4).

### The p38 MAPK signaling pathway: TJs and AJs dynamics

The p38 MAPK signaling pathway participates in the multiple signaling pathway network involved in regulating JAM-B. We have described above how TGF-β2 and TGF-β3 suppress expression of JAM-B via the TGF-β/Smad signaling pathway. Herein, the p38 MAPK signaling pathway also involves in the interleukin-1α (IL-1α) promotion of JAM-B transcription in rat SCs. Activated p38 MAPK phosphorylated the ETS domain transcription factor (Elk-1). Phosphorylation allows Elk-1 to bind on TGIF and proximal Sp1 (pSp1) + E2F motifs. Such interaction will increase Sp1 and NRSF trans-activated *JAM-B* transcription finally^74^. We would like to mention the two other MAPK subfamilies, JNK and ERK here, for their effects on destabilization of JAM-B mRNA transcript via post-transcriptional regulation upon TGF-β3 stimulation in mouse SCs^75^.

Moreover, TGF‐β3 has been found to perturb TJs barrier assembly in the p38 MAPK signaling pathway via a transient increase in phosphorylated p38-MAPK instead of overall p38 MAPK^102,103^. Overexpression of TGF-β3 in primary rat SCs can magnify above damage effect in vitro, with occludin, N-cadherin, and ZO-1 decline^104^. In CdCl_2_-induced adult rat BTB damage, a specific p38 MAPK activity inhibitor SB202190 can blocked loss of ZO-1 and occludin, and thus abolish the damage of TGB-β3 on the AJ and TJ barrier function. It strengthens the physiological importance of the TGF-β3/p38 MAPK signaling pathway in AJ and TJ dynamics^105^.

The event differs when the p38 MAPK signaling pathway and/or the ERK signaling pathway regulate cell junctions upon TGF‐β3 and tumor necrosis factor α (TNF-α) treatment. After adapter CD2-associated protein (CD2AP) binds to TGF-β3 and TGF-β receptor I (TGFβRI) complex, the BTB integrity remains normal, but SCs-GCs adhesion is disrupted reversibly via the activated ERK-signaling pathway in rat SCs. However, when both TAK1-binding protein 1 (TAB1) and CD2AP bind to TGFβR I, the p38 MAPK and ERK signaling pathway are both activated. Not only occludin, ZO-1 at the BTB, but also cadherins at the apical ES and BTB decreases, which leads to disruption of SCs-GCs adhesion and the BTB as well^104^. As for TNF-α, it binds to TNFR1 and/or TNFR2 on rat SCs membrane and activates only p38 MAPK without ERK, downregulating occludin and ZO-1 expression transiently to allow relocation of preleptotene and leptotene spermatocytes crossing the BTB and differentiation of them to pachytene spermatocytes^106^.

### The JNK signaling pathway: TJs and AJs dynamics

Recent evidences strongly support that the JNK signaling pathway contributes to the BTB function and GCs migration. Intercellular adhesion molecule-1 (ICAM-1) is the constitution of BTB and a pivotal regulator in BTB dynamics, which is co-localized with occludin and N-cadherin^6^. After transfected with pCI-neo/ICAM-1 plasmids, the rat SCs overexpressed *ICAM-1* with increase of transepithelial electrical resistance and enhancement of TJs barrier function^107^. TNF-α stimulation on JNK is related to ICAM-1. After secreting from round spermatids, TNF-α binds to the p55 receptors (TNFR1) on mouse SCs membrane, activates the JNK signaling pathway and thus increases ICAM-1 expression^108,109^. Further studies need to focus on whether ICAM-1 overexpression can stabilize TJ dynamics in vivo upon TNF-α-activated JNK pathway.

Furthermore, the JNK signaling pathway will also reduce CdCl_2_-induced BTB disruptive effects in adult rats, which is just contrary to the p38 MAPK signaling pathway. During CdCl_2_-induced BTB disruption, the JNK signaling pathway leads to α_2_-macroglobulin (α_2_-MG) expression, which is a protease inhibitor localized at the SCs-SCs and SCs-GCs interface^110^. Wong et al. used the protein kinase inhibitor 6-dimethylaminopurine which can downregulate α_2_-MG protein level to examine its effect^111^. After 6-dimethylaminopurine pretreatment before CdCl_2_ administration in rat, they observed losing of GCs and flaking of the most seminiferous epithelium in the basement membrane^111^. These evidences reveal the importance of α_2_-MG in inhibiting unwanted proteolysis and maintaining TJs and AJs integrity in defending the CdCl_2_-induced BTB disruption.

### The ERK signaling pathway

#### GCs proliferation and meiosis

Different from the JNK and p38 MAPK signaling pathways, the ERK signaling pathway directly regulates apoptosis, mitosis, and meiosis progression of GCs. In situ hybridization of mouse testis and primary cell culture have confirmed that fibroblast growth factor-4 (FGF-4) expresses only in Sertoli cells throughout the spermatogenic cycle^112^. Hirai et al. investigated that overexpression of FGF-4 in mouse SCs inhibited apoptosis of GCs due to mild hyperthermia. They injected mice with recombinant *FGF-4* adenovirus and then treat them at 43 °C for 15 min after 5 days. Dissection of testis showed fewer sperm count and less testicular weight in response to mild heat treatment than that of control, along with the increase of phosphorylation level of the ERK1/2 in mouse SCs and GCs. It indicates the potential mechanism that FGF-4 prevents GCs from apoptosis and promotes GCs survival via triggering the ERK signaling pathway in SCs and GCs^113,114^.

Furthermore, meiosis of spermatocytes depends on activation of the ERK signaling pathway in co-culture of SCs and pachytene spermatocytes. Godet et al. detected the phosphorylated ERK1/2 in such co-culture. After pre-treatment of MEK1/2 inhibitor U0126, the number of pachytene spermatocytes and secondary spermatocytes declined. But no similar phenomenon emerged in pachytene spermatocytes culture upon U0126 treatment. These different phenomena emphasize the determination of the ERK signaling pathway in SCs for spermatocytes meiosis^115^.

GDNF has been identified as a paracrine factor to promote proliferation and migration, but prevents differentiation of SSCs via binding onto the RET/GFRα1 co-receptors and activating of Ras/ERK1/2 signaling pathway in SSCs^16,28,116^. GDNF expression in mouse SCs can be upregulated via the cAMP/PKA signaling pathway and the Wnt/β-catenin signaling pathway^117,118^, but be downregulated by the Notch signaling pathway^119–122^. Mouse SCs also use the ERK signaling pathway for regulating GNDF expression and thus influencing SSCs niches. During the self-renewal phase of mouse SSCs, the level of GDNF in SCs rises with the activation trend of ERK 1/2 in SCs, which preserves the undifferentiated state of SSCs^123^.

#### SCs proliferation

FSH decides the states of the ERK signaling pathway at a stage-dependent manner in SCs, with each stage activated or inhibited. At 5 days after birth, FSH treatment on isolated rat SCs stimulated MEK-1 activation, and then increased phosphorylation and nucleic relocation level of ERK1/2, the former of which can be eliminated by pre-incubation of SCs with MEK-1 inhibitor PD98059. This way, the expression of cyclin D1 (CCND1) and proliferation rate of the neonatal SCs are promoted. However, SCs maturation stage displays an opposite effect of FSH on the ERK signaling pathway. At 19 days after birth, FSH treatment turns to inhibit the ERK signaling pathway in rat SCs, leaving number of S-phase SCs and protein level of CCND1 less sensitive to FSH stimulation^124^. Similar trends of phosphorylated ERK were also detected in normal mice, though without FSH treatment in vitro, where phosphorylation level of ERK increased until puberty, followed by a decrease during adulthood in wild type mice^125^.

Furthermore, ouabain, which is a mammal adrenal gland cortex-produced endogenous cardiotonic steroid, can induce CCND1 expression and primary rat SCs proliferation accompanied with activation of the ERK signaling pathway^126^. We have addressed the changes in phosphorylated ERK levels is consistent with the proliferation of SCs, so that the periodical rising and falling of the ERK signaling pathway activation are probably closely linked with numbers of SCs and differentiated GCs during testicular development.

#### Lactate and iron supply

FGF-2 utilizes the ERK signaling pathway to regulate transferrin secretion and lactate dehydrogenase (LDH) activity in rat SCs, thus influencing iron and lactate supplies for GCs, respectively. Incubation of rat SCs with U0126 or PD98059 both blocked phosphorylated-ERK-induced transferrin secretion and LDH catalytic activity^127^. Galardo et al. further analyzed the intrinsic molecular mechanism behind these results^128^. There is a CRE-like sequence on the promoter of the transferrin encoding gene and a consensus CRE sequence on the promoter of the *LDH A* gene in rat^129–131^. Treating rat SCs cultures with FGF-2 could increase phosphorylated CREB level, while PD98059 incubation inhibited FGF-2 stimulation on phosphorylated CREB, LDH A, and transferrin uprising level^128^. So CREB may act as the target of ERK1/2 signaling to regulate iron and lactate supplies in SCs.

## Pathways and potential clinical applications of abnormal spermatogenesis

In patients with testicular tumor or infertility, abnormal activity of signaling pathways was observed, including the Wnt signaling pathway, the PI3k/Akt signaling pathway, etc^56,132–137^. We had discussed of the TGF-β/Smad, AMPK, and MAPK signaling pathways in SCs to regulate normal spermatogenesis. There are also clinical studies which revealed the relevance of the three pathways and abnormal spermatogenesis.

Infertility is an emerging worldwide public health issue^138,139^. From 1990 to 2010, the number of infertile couples increased globally, and 48.5 million couples worldwide were disturbed^140^. Among them, approximately 20–70% of cases are owing to the male factor, and at least 30 million men worldwide being diagnosed with infertility according to statistic research in 2015^141^. Abnormal quality and insufficient quantity of sperm are the primary causes of male infertility, most of which are clinically manifested as oligozoospermia, asthenozoospermia, teratospermia, or azoospermia^142^. Azoospermia is classified as obstructive azoospermia and nonobstructive azoospermia^143^, the latter of which is a major course for male infertility and affects 10–15% of infertile men^144^. The microarray analysis on testicular biopsy samples from azoospermic men detected over-activation of the MAPK signaling pathway in SCs^145^. For azoospermic patients, Sertoli cell-only syndrome affects 26.3–57.8% of them, whose testicular histology biopsies shows no germ cells and only Sertoli cells in the seminiferous tubules^146^. In testicular biopsies from nonobstructive azoospermia patients with Sertoli cell-only syndrome, BMP4, TGF-β receptor II (TGFβRII), and Smad2 are more highly expressed^57,70^. As for the AMPK signaling pathway, studies in SCs of infertile humans are insufficient. However, number of pups per litter in SC-α1AMPK-cKO mice did decrease by 25%, accompanied with disturbed cell junction dynamics^86^. Thus, for the purpose of elucidating the molecular basis and developing therapeutic options for azoospermia therapy, the causes of azoospermia deserve more attention in the future, especially from the perspective of the TGF-β/Smad, AMPK, and MAPK signaling pathways in SCs^147–150^.

Testicular cancer is a common malignancy which can cause infertility and death in men^151^. In the year of 2018, the worldwide estimated number of new cases of testicular cancer at all ages reached 71,105 according to International Agency for Research on Cancer^152^. Testicular tumors can be classified into germ cell tumors, sex cord–stromal tumors, mixed germ cell/sex cord–stromal tumors, and lymphomas^153^. Sex cord-stromal tumors consist of Sertoli cell tumors, Leydig cell tumors, granulosa cell tumors, and unclassified tumors^133,154^. Testicular cancer development is potentially linked with the TGF-β/Smad signaling pathway, especially when the BMP signaling SMADs (BR-SMADs) participate. Smad4, the Co-Smad in the TGF-β/Smad signaling pathway, may serve as a key mediator in Leydig cell adenomas. When Smad4 was conditionally knocked out in mouse Sertoli cells and Leydig cells, 87.5% of the mutant mice exhibited Leydig cell adenomas at 56–62 weeks of age^64^. After the BR-SMADs (Smad1, 5) in mice SCs is deleted via tissue-specific ablation, all male Smad1/Smad5 KO mice (14 samples) developed Sertoli-Leydig tumors after 28 weeks of age with 100% metastases to lymph and peritonea, implicating the role of the BR-SMAD signaling pathway as a tumor suppressor in testis^155^.

## Conclusions and perspectives

In present, the relationship between signaling pathways, infertility and tumorigenesis in SCs still remains unknown. However, various hormones, cytokines or proteins have been indicated to express differently in SCs if abnormal spermatogenesis occurs^156^. For instance, FSH suppresses Sertoli cell tumor progression during the 1st or 2nd week after birth, which is the first wave of spermatogenesis in inhibin α-KO mice^157^. Since these signaling molecules are often involved in multiple signaling pathways in SCs or regulated by various signaling pathways^158,159^, identifying the determining signaling pathway that controls abnormal spermatogenesis is the first step to study the causes of abnormal spermatogenesis, progression of testicular cancer, and infertility^160^. These basic researches may facilitate diagnostics and therapeutics for testicular cancer and infertility, as well as development of targeted drugs, and all these advances will reduce cancer mortality and infertility morbidity in the future.
